# Predicting Reading From Behavioral and Neural Measures – A Longitudinal Event-Related Potential Study

**DOI:** 10.3389/fpsyg.2021.733494

**Published:** 2021-11-30

**Authors:** Aleksandra K. Eberhard-Moscicka, Lea B. Jost, Moritz M. Daum, Urs Maurer

**Affiliations:** ^1^Department of Psychology, University of Zurich, Zurich, Switzerland; ^2^Perception and Eye Movement Laboratory, Department of Neurology and BioMedical Research, Inselspital, Bern University Hospital and University of Bern, Bern, Switzerland; ^3^Department of Neurology, Inselspital, Bern University Hospital, Bern, Switzerland; ^4^Department of Neuroscience and Movement Science, University of Fribourg, Fribourg, Switzerland; ^5^Department of Psychology, The Chinese University of Hong Kong, Shatin, Hong Kong SAR, China; ^6^Brain and Mind Institute, The Chinese University of Hong Kong, Shatin, Hong Kong SAR, China

**Keywords:** N1 print tuning, MMN, audio-visual integration, EEG, ERP, reading, development, longitudinal

## Abstract

Fluent reading is characterized by fast and effortless decoding of visual and phonological information. Here we used event-related potentials (ERPs) and neuropsychological testing to probe the neurocognitive basis of reading in a sample of children with a wide range of reading skills. We report data of 51 children who were measured at two time points, i.e., at the end of first grade (mean age 7.6 years) and at the end of fourth grade (mean age 10.5 years). The aim of this study was to clarify whether next to behavioral measures also basic unimodal and bimodal neural measures help explaining the variance in the later reading outcome. Specifically, we addressed the question of whether next to the so far investigated unimodal measures of N1 print tuning and mismatch negativity (MMN), a bimodal measure of audiovisual integration (AV) contributes and possibly enhances prediction of the later reading outcome. We found that the largest variance in reading was explained by the behavioral measures of rapid automatized naming (RAN), block design and vocabulary (46%). Furthermore, we demonstrated that both unimodal measures of N1 print tuning (16%) and filtered MMN (7%) predicted reading, suggesting that N1 print tuning at the early stage of reading acquisition is a particularly good predictor of the later reading outcome. Beyond the behavioral measures, the two unimodal neural measures explained 7.2% additional variance in reading, indicating that basic neural measures can improve prediction of the later reading outcome over behavioral measures alone. In this study, the AV congruency effect did not significantly predict reading. It is therefore possible that audiovisual congruency effects reflect higher levels of multisensory integration that may be less important for reading acquisition in the first year of learning to read, and that they may potentially gain on relevance later on.

## Introduction

Developmental dyslexia is usually identified after a child has started to learn to read at school. This delayed identification comes with a delay of supportive measures and an increase of the reading deficits compared to typically developing children. Given that dyslexia is thought to arise from preexisting neurocognitive deficits, there is great interest in finding longitudinal predictors of reading development that may be used for the early identification of dyslexia. In addition to behavioral predictors, such as phonological deficits, cognitive neuroscience research also identified several unimodal neural measures that may improve longitudinal prediction of reading development compared to behavioral measures alone (e.g., Hoeft et al., 2007; Maurer et al., 2009). Given the multimodal nature of reading, and particularly the importance of print-to-sound mapping (Ehri and Wilce, 1985), the question arises whether neural measures of audiovisual integration can further improve the prediction of reading development. In addition to the potential practical significance of reading predictors, they are also theoretically relevant, as they point to processes that are particularly important for reading acquisition at certain stages of reading development and may further be used to guide age-specific interventions.

Several longitudinal studies have examined early behavioral predictors of later reading abilities. To date, the best behavioral predictors of reading outcome in alphabetic languages are recognized to be rapid automatized naming (RAN; i.e., the ability to quickly and accurately name a series of items, e.g., pictures or familiar objects), phonological awareness (the ability to identify and manipulate the sound units of a word), letter knowledge and vocabulary (Juel, 1986; Wolf, 1986; Wimmer et al., 1991; Bowey, 1995; Wagner et al., 1997; de Jong and van der Leij, 1999; Catts et al., 2001; Pennington and Lefly, 2001; Schatschneider et al., 2004; Lepola et al., 2005; Puolakanaho et al., 2007; Georgiou et al., 2008; Torppa et al., 2012; Brem et al., 2013). The relative importance of these cognitive skills may change depending on the orthographic depth of a particular writing system, with rapid naming being a more important predictor than phonological awareness in more transparent languages like Finnish, Italian or German (Moll et al., 2014; Zoccolotti et al., 2014; Schmalz et al., 2015). Based on such findings, behavioral tests have been developed to assess the risk for developing dyslexia shortly before school entry (e.g., Jansen et al., 2002). However, despite encouraging results, considerable variance in reading development remained unexplained, and the question arose whether prediction could be improved by measuring neurocognitive processes that underly phonological and orthographic processing (Vandermosten et al., 2015) more directly.

Several neuroimaging studies have provided evidence that concurrent reading skills or future reading development can be predicted based on either spatially (e.g., Hoeft et al., 2007, 2011; Raschle et al., 2011, 2012; Karipidis et al., 2018) or temporally sensitive (e.g., Maurer et al., 2009) neural measures. While both approaches are theoretically relevant, temporally sensitive EEG measures have a practical advantage due to their easier and less expensive application, and therefore the focus of the following literature review is on EEG studies. In EEG studies, two neural measures have been mainly discussed as possible early predictors of later reading outcome. One of them being a visual, negative component of the event-related potential (ERP), known as N170, N1 or N1 print tuning (Bach et al., 2013; Brem et al., 2013), and the other one being a negative component of the auditory ERP, namely mismatch negativity (MMN, Maurer et al., 2009). However, to our knowledge the predictive values of these two measures have not been tested in the same study with the same children.

The N1 component of the visual ERP peaks at around 150–250 ms after stimulus presentation and is characterized by posterior negativity and fronto-central positivity, thought to be generated by sources in bilateral occipito-temporal regions (e.g., Bentin et al., 1999; Tarkiainen et al., 1999; Brem et al., 2005, 2009; Parviainen et al., 2006; Maurer et al., 2007). Although elicited by visual stimuli in general, the N1 is enhanced for expertise-related stimuli compared to low-level visual control stimuli (Rossion et al., 2003). In the area of reading, words that are presented visually elicit a larger N1 than symbol strings (Bentin et al., 1999; Maurer et al., 2005a,b; Brem et al., 2006) or false-font strings (Brem et al., 2010; Hasko et al., 2013; Eberhard-Moscicka et al., 2014, 2016), an effect that has been called N1 print tuning, and that is thought to reflect visual expertise for letter strings (Maurer et al., 2005b, 2006). This neural specialization for print is not only present in adult expert readers (Maurer et al., 2005a; Brem et al., 2006; Mahé et al., 2012), but also in beginning readers (Eberhard-Moscicka et al., 2014; Zhao et al., 2014) and most strikingly already in illiterate kindergarten children after only a short grapheme–phoneme training (Brem et al., 2010). Print tuning has been shown to be reduced in children with dyslexia (Maurer et al., 2007) and to correlate with concurrent reading skills (Eberhard-Moscicka et al., 2014). Next to this visual expertise account, there is another account of N1 print tuning that is believed to reflect the print-to-sound mapping (Brem et al., 2013). This account has previously been used to explain the often reported left-lateralization of the N1 print tuning (Maurer and McCandliss, 2007). Importantly, previous studies pointed toward the predictive value of the N1 print tuning that can serve as an early predictor of the later reading outcome (Brem et al., 2010; Bach et al., 2013).

The MMN is a negative component in a difference ERP between deviant and standard auditory stimuli that peaks at around 100–250 ms at fronto-central electrodes. The fronto-central negativity is accompanied by a positivity at temporal/mastoid electrodes reflecting auditory sources and possibly an involvement of frontal cortices (for a review, see Alho, 1995). The MMN is evoked automatically in an oddball condition where infrequent deviant stimuli are embedded among frequently occurring standard stimuli and is thought to measure sensory memory (Näätänen and Alho, 1997; Näätänen et al., 2005). It is independent of attention, which makes it a successful tool to investigate phoneme specialization in young children (for a review, see e.g., Näätänen et al., 2007) who are easily distracted or sometimes difficult to motivate to participate in experimental tasks. To date, MMN has been widely used in research with preschoolers (e.g., Maurer et al., 2003; Lee et al., 2012; Lovio et al., 2012) and school-age children (e.g., Kraus et al., 1999; Cheour et al., 2000; Maurer et al., 2009; Datta et al., 2010; Jost et al., 2015). The MMN has been shown to be reduced in dyslexia for speech and non-speech stimuli (e.g., see Gu and Bi, 2020 for a recent meta-analysis). In preschool children, the amplitude and the degree of the left-lateralization of the late MMN improved the prediction of reading ability over behavioral measures, but lateralization was the only measure capable of predicting long-term reading outcomes in fifth grade (Maurer et al., 2009). Prospective prediction of reading or reading-related skills was also obtained from measures of auditory processing in infants (Molfese, 2000; Lyytinen et al., 2004; Guttorm et al., 2010).

While N1 and MMN measures have been shown to be able to prospectively predict reading development, they are unimodal measures that do not reflect an essential aspect of learning to read, which is the linking of visual and auditory information (Blomert, 2011). It is believed that this bimodal grapheme–phoneme integration is an emergent property of learning to read which may develop inadequately in dyslexic children (Blau et al., 2010) and adults (Blau et al., 2009), presumably due to lacking specialization at the neuroanatomical level. Brain regions that are believed to play a role in the binding of grapheme–phoneme pairs have been located to temporal and occipital brain areas (Raij et al., 2000; van Atteveldt et al., 2004). One way of investigating the audiovisual (AV) integration is by comparing the neural response of incongruent and congruent audiovisual stimuli, the so-called AV congruency effect. This AV congruency effect has been demonstrated at the more basic level of letter-speech sound pairs (van Atteveldt et al., 2007; Doehrmann and Naumer, 2008; Karipidis et al., 2017) but also at the level of word-speech sound pairs (Jost et al., 2013). While theoretical arguments point to the potential use of AV integration measures for predicting reading, only few studies have been conducted so far. In one study, an ERP congruency effect after an artificial letter training in kindergarten improved prediction of poor reading in a small sample of children who were followed up half a year after the onset of reading training at school (Karipidis et al., 2018). In our own study, we found no clear association between audiovisual integration and concurrent reading fluency in first grade children (Jost et al., 2013), thus leaving it an open question whether such an association would emerge only later in the course of reading acquisition. Furthermore, as previous studies indicated the predictive power of basic unimodal visual (i.e., N1 print tuning; Bach et al., 2013; Brem et al., 2013) and auditory (i.e., MMN; Maurer et al., 2009) neural measures, the question arises as to the relative contribution of unimodal visual and auditory measures and a bimodal measure of audiovisual integration regarding their prediction of the later reading outcome.

Thus, the current study made use of behavioral and neural measures from children tested in first grade (previously reported in Jost et al., 2013, 2015; Eberhard-Moscicka et al., 2014, 2016) to predict reading skills of the same children who were followed up in fourth grade as part of the current study. Unlike some of the previously used EEG indices (Eberhard-Moscicka et al., 2014, 2016), this study employed the whole-scalp topographic approach (as also reported in Jost et al., 2013, 2015) to account for different scalp-distribution patterns across all the three neural measures tested. Moreover, to obtain the typical MMN topography (i.e., fronto-central negativity and lateral/mastoid positivity, e.g., Maurer et al., 2003; Kujala et al., 2007; Näätänen et al., 2007; Zevin et al., 2010) additional filter settings (cf. Jost et al., 2015) were applied to the MMN data. The goals of the study were to investigate: How well do behavioral measures collected at the end of first grade predict the reading outcome at the end of fourth grade (aim 1); How much of the variance in reading at the end of fourth grade can be attributed to all the three neural measures from first grade (aim 2); Whether neural measures add to the prediction over behavioral measures (aim 3).

## Materials and Methods

### Participants

We report data of 51 native (Swiss-)German-speaking children (21 girls and 30 boys; 4 left-handed, 5 dyslexics, i.e., below 10th percentile). Children were tested longitudinally; the first assessment took place after 1 year of formal reading instruction (i.e., at the end of first grade, mean age 7.6 years, range 6.7–8.5 years), whereas the second assessment took place at the end of fourth grade (mean age 10.5 years, range 9.6–11.2 years). From an original group of 70 children, seven dropped out of the study, one transferred to another school, two needed to repeat a grade, six were excluded due to a low number of accepted trials in either the N1 task (four children were below 26 trials) or in the MMN task (two children were below 70 trials), and three participants were above three standard deviations in the Global Field Power (GFP) of the time window of interest in either of the three EEG tasks. All subjects had normal or corrected-to-normal vision, and every child had an estimated non-verbal IQ equal or above 80 [i.e., not more than 1.333 *SD* below the normative mean in HAWIK-IV (*M* = 100, *SD* = 15), subtest: block design, Petermann and Petermann (2010), corresponding to the English version of the Wechsler Intelligence Scale for Children]. The study protocol was approved by the local ethics committee. Consent was obtained orally from children and in written form from their parents. Moreover, children’s parents filled out a background questionnaire screening for a history of neurological diseases and psychiatric disorders.

### Procedure

In first grade, all the children participated in a behavioral and an EEG session (previously reported in Jost et al., 2013, 2015; Eberhard-Moscicka et al., 2014, 2016), while in fourth grade they participated in a behavioral session only. At both time points (i.e., at the end of first and fourth grades) the behavioral session lasted about 1.5 h and took place either at schools (in a separate room provided by schools), at the Department of Psychology at the University of Zurich or at participants’ homes. The EEG session was administrated using one of two identical portable EEG systems (Electrical Geodesics, Inc., EGI). The recording was approximately 3.5 h long and was administered either in a separate room provided by schools or in the EEG laboratory at the Department of Psychology at the University of Zurich. Before using a room at the schools, a standard quality check was applied to ensure the absence of 50 Hz noise. As a compensation for the participation in the study, every child received a written report about his/her reading skills and a book voucher of 40 CHF at the first assessment at the end of first grade and of 30 CHF at the second assessment at the end of fourth grade.

### Behavioral Session

During the behavioral assessment, the child was seated opposite the experimenter and performed a set of cognitive tasks. All the tasks were rehearsed according to test guidelines to make sure that every child understood the instructions. The measures collected during the behavioral session assessed different aspects of German language processing. In first and fourth grades, measures of sentence- and word-reading fluency (Mayringer and Wimmer, 2005; Landerl et al., 2006; Moll and Landerl, 2010) were collected. Next to the reading fluency measures also RAN (Landerl, 2001; Landerl et al., 2013), phonological awareness (Stock et al., 2003), vocabulary, auditory memory span and block design as a measure of non-verbal IQ (Petermann and Petermann, 2010), as well as spelling (Moll and Landerl, 2010) were assessed in first grade (see Table 1 for a detailed list of subtests and Supplementary Material T1 for bivariate correlations between behavioral measures in first grade). The spelling task proved to be too difficult for the first graders, hence could not be considered in further analyses.

**TABLE 1:** Behavioral and neural measures used in the regression approach.

	*M (SD)*	Correlations			
Measures		Reading fluency in fourth grade	N1 print tuning in first grade (GFP)	Filtered MMN in first grade (GFP)	AV congruency in first grade (GFP)
**Reading fluency in fourth grade**					
SLRT I word-reading (correct per 1 min)	107.7(29.9)	1.00	0.38^2^	–0.22	–0.21
SLRT-II word-reading (correct per 1 min)	75.6(19.4)				
SLRT I text-reading (correct per 1 min)	126.9(30.4)				
SLS sentence-reading (correct per 3 min)	50.0(10.7)				

**Reading fluency in first grade**					
SLRT I word-reading (correct per 1 min)	35.5(20.9)	0.56^1^^*^	0.34^3^	–0.14	–0.11
SLRT-II word-reading (correct per 1 min)	30.3(15.6)				
SLRT I text-reading (correct per 1 min)	47.9(33.2)				
SLS sentence-reading (correct per 3 min)	18.8(9.4)				

**RAN in first grade**					
RAN one syllable animals naming (time in sec)	69.5(18.2)	−0.55^2^	–0.21	0.10	0.08
RAN three syllable animals naming (time in sec)	90.2(26.8)				
RAN lower case letter naming (time in sec)	39.7(9.8)				
RAN digit naming (time in sec)	40.7(12.1)				

**Phonological processing in first grade**					
BAKO phoneme deletion (correct items/max: 7)	4.5(1.7)	0.37^2^	–0.06	–0.23	–0.08
BAKO pseudoword segmentation (correct items/max: 8)	4.8(1.5)				

**Vocabulary in first grade**					
HAWK-IV, vocabulary (raw score)	26.7(6.1)	0.42^2^	0.09	–0.00	0.02

**Block design in first grade**					
HAWK-IV, block design (raw score)	33.5(11.0)	0.05	0.06	–0.16	–0.08
Auditory memory span in first grade					
HAWIK-IV, digit span backward (raw score)	5.8(1.2)	0.30^3^	0.18	–0.19	–0.10
HAWIK-IV, digit span forward (raw score)	6.5(1.0)				

^1^*p* < 0.001; ^2^*p* < 0.01; ^3^*p* < 0.05; asterisk depicts significant *p < 0.005 Bonferroni corrected value.

*Standard score: vocabulary 11.16 (2.44), block design 12.12 (3.00), digit span (backward and forward) 10.33 (2.07).*

The measure of reading fluency in fourth grade was based on average scores of four z-transformed tests of word, text, and sentence reading (see also Table 1). Scores for correct words per minute were computed for the two subtests of the Lese- und Rechtschreibtest (Landerl et al., 2006) and one subtest of the Salzburger Lesetest II (Moll and Landerl, 2010). The score for correct sentences per minute was computed based on the Salzburger Lesescreening 1-4 (Mayringer and Wimmer, 2005).

### EEG Session

During the EEG recording, children were seated 80 cm away from the computer screen. Every child performed two unimodal (i.e., N1 and MMN) and one bimodal (i.e., AV) EEG tasks (described below) that were presented in a pseudo-randomized order. To avoid fatigue, children were allowed to take breaks between experiments and compliance during the experiments was monitored by means of a digital camera. Before every experiment began, children were instructed on task demands. Additionally, as opposed to the passive MMN task, for the active N1 and AV tasks children performed a practice experimental run that lasted about 1 min.

#### EEG Tasks

##### Visual one-back N1 task

**FIGURE 1:**
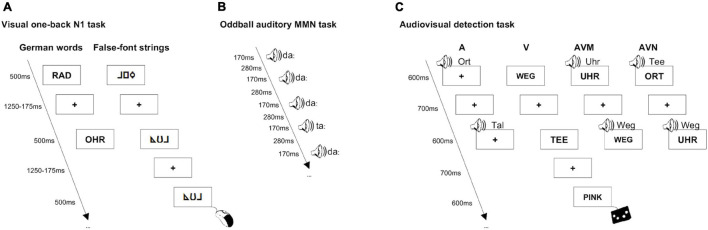
First grade children performed two unimodal and one bimodal EEG tasks that were presented in a pseudo-randomized order. In the visual one-back N1 task they were viewing German words and false-font strings and were instructed to press a mouse button for immediate repetitions **(A)**. In the oddball auditory MMN task they were watching a silent cartoon while in the background they were presented with repetitive standard “da” and rare deviant “ta” sound stimuli **(B)**. In the audiovisual detection task German words were presented either in the auditory, visual, audiovisual matching or audiovisual non-matching mode and children were asked to press the response pad button whenever they saw or heard the target word “PINK” **(C)**.

The visual N1 task (see also Eberhard-Moscicka et al., 2014, 2016) assessing specialization for print took about 20 min. In this task, children were presented with familiar German words (high frequency of occurrence in the textbooks of children aged 6–8, *M* = 161.86/Mio, ChildLex Lexical Database, Schroeder et al., 2015), unfamiliar false-font strings matched to the letters appearing in German words (false-font characters were designed for the purpose of this study where each alphabetical letter had its unique false-font correspondent), English words and pseudowords and were asked to press a mouse button for immediate repetitions (Figure 1A). English word and pseudoword stimuli are not part of this study, hence will not be described in detail here (for a detailed description we refer the reader to Eberhard-Moscicka et al., 2016). Due to the limited number of English words that we expected children to know at the follow-up session (Eberhard-Moscicka et al., 2016), we limited the number of items per condition to 14. The 14 stimuli per condition were repeated six times (84 stimuli per condition) and presented in six blocks (the order of conditions was counterbalanced). In each condition, 12 immediate repetitions serving as targets were presented. To be consistent with previous studies (e.g., Maurer et al., 2005a,b, 2006, 2007), the stimuli were presented in a block design and the block order was counterbalanced across subjects. Stimuli were presented in black (Arial, bold, font size 28, uppercase letters) and appeared in the middle of a white rectangular box (85 mm × 47 mm) in the center of a gray background. Each stimulus was presented for 500 ms and was followed by a mean inter-stimulus interval of 1500 ms (jittered between 1250 and 1750 ms). The stimuli were matched for string length and contained 3.9 letters/false-font characters on average (range: 3–5; average length and height: 31.9 mm × 7 mm). In addition, German words, pseudowords and English words were matched for number of letters, frequency of letters and number of syllables. In this paper, we focus on the N1 print tuning effect in the native German language, i.e., the difference between German words and false-font strings, thus only data of these experimental stimuli will be analyzed and discussed here.

##### Oddball auditory mismatch negativity task

The auditory MMN task (see also Jost et al., 2015) assessing phoneme specialization took approximately 15 min. In this passive task, children were asked to avoid motion and watch a silent cartoon while in the background they were presented with repetitive standard and rare deviant sound stimuli. The phonemes presented were one standard “da” and two deviants “ta” (a common phoneme in the native German language) and “tha” (a common phoneme in the non-native English language, not part of this study, hence not discussed here, for details we refer the reader to Jost et al., 2015). The natural speech stimuli were matched for vowel onset and duration, as well as for maximal intensity (Praat software, Boersma, 2001) and were presented in a traditional oddball paradigm where the deviant stimuli occurred 9.4% of the time. The stimuli were presented binaurally through speakers placed in front of the subject and next to the laptop playing a silent cartoon. A total of 1600 standard (“da”) and 300 deviant (150 deviant “ta” and 150 deviant “tha”) stimuli were presented for 170 ms and followed by a 280 ms inter-stimulus interval (Figure 1B). Stimulus order was pseudo-randomized so that at least two standards were played between two deviants. Here, we focus on the MMN in the native German language, that is, the difference between deviant “ta” and standard “da,” hence only data of these experimental stimuli will be analyzed and discussed.

##### Audiovisual detection task

The audiovisual (AV) detection task (see also Jost et al., 2013) assessing the integration of visual and spoken words lasted for about 24 min. In this task children were asked to respond to a rare (9%) target word “PINK” by pressing a response pad button. The stimuli presented were 10 familiar German words (high frequency of occurrence in the textbooks of children aged 6–8, *M* = 95.37/Mio, ChildLex Lexical Database, Schroeder et al., 2015), 10 unfamiliar English words (pronunciation according to German grapheme–phoneme correspondence rules and phonetic inventory) and 10 unfamiliar English words (pronunciation not according to German grapheme–phoneme correspondence rules or phonetic inventory). Similarly to the visual one-back N1 task, the number of unique stimuli was limited to 10 per word list due to the limited sample of English words that the children were expected to know at the follow-up session and due to the German word stimuli and English word stimuli matching procedure. English words are not part of this study, hence will not be discussed in more detail (for details we refer the reader to Jost et al., 2013). Stimuli were presented either in the auditory (A), visual (V) or audiovisual (AV) mode (Figure 1C). The bimodal stimuli were either matching (AVM) or non-matching (AVN). As there was only one target word, the bimodal targets were always matching. Same as for the visual one-back N1 task, the visual stimuli were presented in black (Arial, bold, font size 28, uppercase letters) and appeared in the middle of a white rectangular box (85 mm × 47 mm) in the center of a gray background. The auditory stimuli, spoken by a German-English bilingual male speaker, were scaled to the same length (Praat software, Boersma, 2001). As such, visual as well as auditory stimuli were presented for 600 ms and were followed by a mean inter-stimulus interval of 700 ms. To avoid fatigue, the experiment was divided into two parts (each about 12 min) and children were allowed to take a short break after 6 min of the task. 80 trials were presented for each of the 12 stimulus types (4 modalities × 3 word types). Every word was presented 24 times in the visual (8 unimodal V, 8 bimodal matching, 8 bimodal non-matching) and 24 times in the auditory modality (8 unimodal A, 8 bimodal matching, 8 bimodal non-matching). Given the overlap in the audiovisual matching condition, there were 40 trials where the same word appeared either in the visual or auditory modality. As such, a total of 960 word stimuli and 96 target stimuli were presented in a block design (cf. Kronschnabel et al., 2013; Karipidis et al., 2017; block order was counterbalanced across subjects) in either of the four different stimulus conditions (i.e., A, V, AVM, and AVN). The stimuli were matched for string length and contained 4.4. letters on average (range: 3–7; average length and height: 35.9 mm × 7 mm). In this paper, we focus on the AV congruency effect in the native German language, i.e., the difference between the AVN and AVM German word stimuli, thus only data of these experimental stimuli will be analyzed and discussed here.

#### EEG Recording and Processing

Continuous 128-channel EEG (HydroCel GSN, EGI NA 300 amplifier) was recorded using one of the two identical portable EGI systems. EEG was recorded against the Cz reference, at a sampling rate of 250 Hz, with high- (0.1 Hz) and low-pass (100 Hz) filter settings. As modern high-input impedance amplifiers and their accurate digital filters for power noise provide excellent EEG signal collection even at higher electrode impedances (Ferree et al., 2001), the electrode impedance was kept below 50 kΩ (cf. Maurer et al., 2005a; Franklin et al., 2007; Rihs et al., 2007; Hämäläinen et al., 2015; Karipidis et al., 2017). The raw data of the two unimodal EEG experiments (i.e., N1 and MMN tasks) was preprocessed using BESA software (including eye blink correction, MEGIS Software, Gräfelfing, Germany, for details see also Eberhard-Moscicka et al., 2014; Jost et al., 2015), while the raw data of the bimodal AV task was preprocessed with Vision Analyzer software (including eye blink correction, Brain Products GmbH, for details see also Jost et al., 2013). Apart from filter settings (see below), the remaining preprocessing steps were identical for all the three EEG experiments, i.e., after channels with extensive artifacts were spline interpolated, the continuous EEG was corrected for eye blinks and trials with artifacts exceeding the max-min difference of 180 μV in any channel were automatically excluded before averaging. For the N1 and AV tasks, the corrected files were digitally low- (30 Hz) and high-pass filtered (0.3 Hz). To obtain the typical MMN topography (i.e., fronto-central negativity and lateral/mastoid positivity, e.g., Maurer et al., 2003; Kujala et al., 2007; Näätänen et al., 2007; Zevin et al., 2010) the EEG-data of the MMN task were digitally low-pass filtered with 30 Hz and high-pass filtered with 3 Hz (hereafter referred to as filtered MMN), as described in Jost et al. (2015). The data was further segmented (−150 ms prior and 850 ms following the stimulus onset) and transformed to the average reference (Lehmann and Skrandies, 1980). The recording reference was used as an additional electrode for further data processing. Including and following the average reference step, the ERPs of all the three experimental tasks were further pre-processed in Vision Analyzer Software. Furthermore, the ERPs were corrected for the amplifier delay of 8 ms (induced by the anti-alias filters of EGI NA300 amplifiers with the current sampling rate; for details see Update to Advisory Notice, 26 November 2014, Electrical Geodesics Inc.; cf. Pegado et al., 2014) and a constant stimulus release delay of 20 ms for the N1 and AV tasks and 24 ms for the MMN task. In the final pre-processing step, the ERPs of all conditions of interest (i.e., German words, and false-font strings for the N1 task, standard “da” and deviant “ta” for the MMN task, as well as AVN German words and AVM German words for the AV task) were averaged separately for each experimental task, after target stimuli of the N1 and AV tasks were automatically excluded. Difference ERPs between conditions of interest (i.e., German words – false-font strings for the N1 task, deviant “ta” – standard “da” for the MMN task and AVN German words – AVM German words for the AV task) were computed, before individual grand averages were calculated.

#### EEG Analysis

**FIGURE 2:**
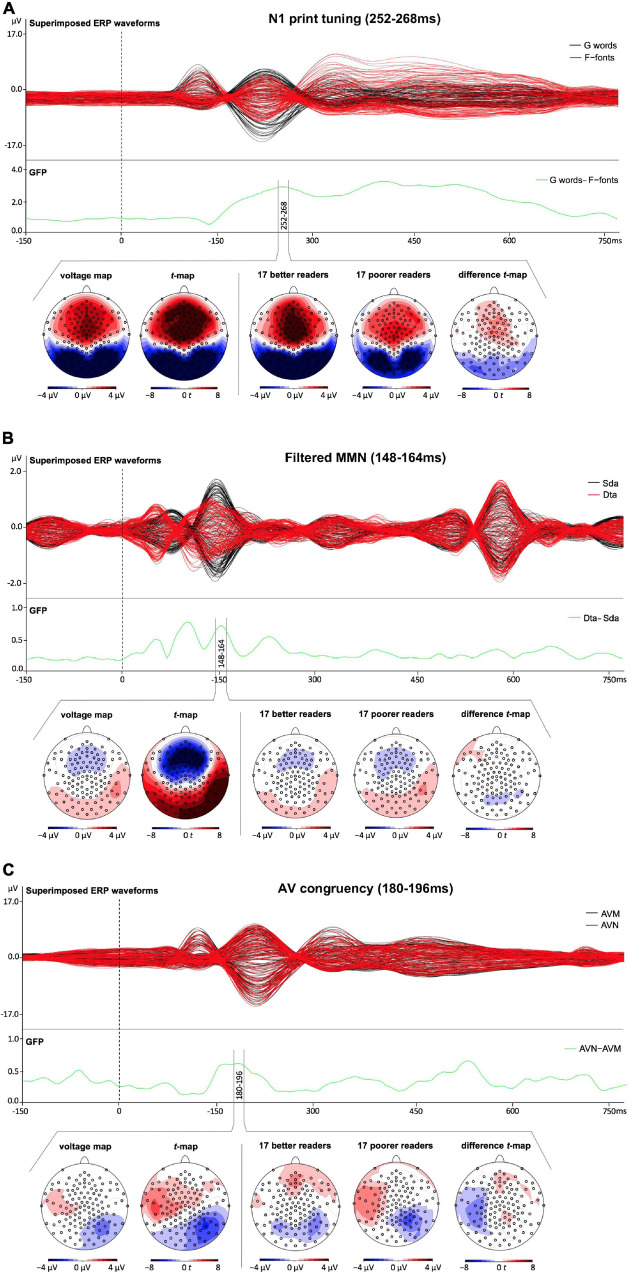
Superimposed event related potential (ERP) waveforms for all the three experimental tasks as well as voltage maps and corresponding *t*-maps across all children and difference *t*-maps of the time segments of interest for the lowest third of poorer and the highest third of better readers. The green line corresponds to the GFP measure of the effects of interest. **(A)** Visual one-back N1 task (black lines correspond to German words and red lines to False-font strings). The green line corresponds to the GFP measure of the effects of interest, i.e., N1 print tuning – indexed by the difference between German words and false-font strings. **(B)** Oddball auditory MMN task (black lines correspond to standard “da” and red lines to deviant “ta” stimuli). The green line corresponds to the GFP measure of the effects of interest, i.e., filtered MMN – indexed by the difference between deviant “ta” and standard “da” stimuli. **(C)** Audiovisual detection task (black lines correspond to audiovisual matching and red lines to audiovisual non-matching German words). The green line corresponds to the GFP measure of the effect of interest, i.e., AV congruency effect - indexed by the difference between audiovisual matching and audiovisual non-matching German words.

We investigated N1 print tuning (indexed by the difference between German words and false-font strings), filtered MMN (indexed by the difference between deviant “ta” and standard “da”) and AV congruency effects (indexed by the difference between AVN German words and AVM German words). The time windows of interest were equally long for all the three EEG tasks (i.e., five time points) and were based on the GFP peaks (i.e., peak ± two time points) of the effects of interest (i.e., N1 print tuning: 252–268 ms, filtered MMN: 148–164 ms, and AV congruency: 180–196 ms, see Figure 2). The chosen time windows coincide with previous studies (N1 print tuning: e.g., Maurer et al., 2006, 2007; Brem et al., 2010, 2013; Araújo et al., 2012; Eberhard-Moscicka et al., 2014, 2016; MMN: e.g., Näätänen et al., 2004; Froyen et al., 2008; Jost et al., 2015; Justen and Herbert, 2018; and AV congruency: e.g., Jost et al., 2013; Karipidis et al., 2017). Given that the aim of this paper was to investigate the early basic processes; early time windows were chosen for all the three neural measures (the analysis on the late AV congruency effect is reported in the Supplementary Material A2). The measure used in the analyses was global field power (GFP; Lehmann and Skrandies, 1980). This whole-scalp topographic measure appears best suited in a study combining different neural measures that follow different scalp-distribution patterns. The GFP represents the spatial standard deviation of the electric field at the scalp (Lehmann and Skrandies, 1980) and has the advantage of being reference-independent (Michel et al., 2004), and thus making it more comparable to the results of previous studies (e.g., Zevin et al., 2010; Jost et al., 2013, 2015).

### Statistical Analysis

Multiple regression analyses were run to predict whether behavioral and neural measures collected at the end of first grade contributed to the explained variance in the reading outcome in fourth grade (aims 1 and 2). A stepwise multiple regression was run to explore if neural measures can improve prediction over behavioral measures (aim 3). All the steps are detailed in the Results section.

## Results

### How Much Variance in Fourth Grade Reading Can Be Explained by the First Grade Behavioral Measures?

**FIGURE 3:**
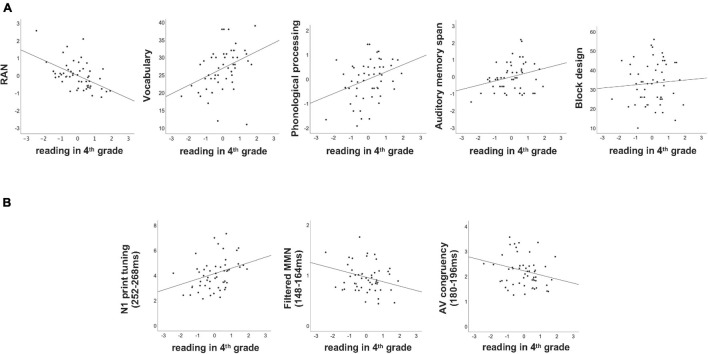
Scatterplots of behavioral **(A)** and neural **(B)** measures collected at the end of first grade and the reading outcome tested at the end of fourth grade. Reading in fourth grade is shown on the *x*-axis (z-transformed), while the behavioral and neural measures are plotted on the *y*-axes (see Table 1 for the units).

Multiple regression was run to explore how much variance in reading in fourth grade can be predicted by the five behavioral measures collected at the end of first grade. Overall, more than 46% of the entire variance in reading at the end of fourth grade could be attributed to the behavioral measures collected at the end of first grade [*F*(5,45) = 7.925, *p* < 0.001, *R*^2^ = 0.468]. Importantly, while RAN (*p* = 0.002), block design (*p* = 0.006) and vocabulary (*p* = 0.007) significantly contributed to the explained variance in reading, auditory memory span and phonological processing were not significant (both *p*’s > 0.221; see also Table 2 and Figure 3A).

**TABLE 2:** Multiple regression analyses (method enter).

Measures	Reading fluency (fourth grade)	*B*	*SE B*	β
Behavioral (first grade)	Constant	–0.34	0.54	
	RAN	–0.50	0.15	−0.42^1^
	Block design	–0.03	0.01	−0.40^2^
	Vocabulary	0.06	0.02	0.37^2^
	Auditory memory span	0.18	0.14	0.16
	Phonological processing	0.17	0.15	0.16

Neural (first grade)	Constant	0.05	0.70	
	N1 print tuning	0.29	0.10	0.40^1^
	Filtered MMN	–0.88	0.43	−0.27^2^
	AV congruency	–0.17	0.20	–0.11

*^1^*p* < 0.005, ^2^*p* < 0.05.*

### Topographic Distribution of the Basic Unimodal and Bimodal Neural Measures

Both unimodal measures of N1 print tuning and filtered MMN showed typical topographic distributions, with N1 print tuning showing a posterior negativity and fronto-central positivity and filtered MMN showing fronto-central negativity and lateral/mastoid positivity (Figure 2). AV congruency showed left fronto-temporal positivity and right occipito-temporal negativity (Figure 2). While the measures of N1 print tuning and filtered MMN were highly significant at multiple occipito-temporal [N1: *t*_(max)_ = −12.70, *p* < 0.001; MMN: *t*_(max)_ = 9.66, *p* < 0.001] and fronto-central electrodes [N1: *t*_(max)_ = 10.52, *p* < 0.001; MMN: *t*_(max)_ = −9.25, *p* < 0.001], AV congruency showed weaker effects, nevertheless still highly significant at right occipito-temporal electrodes [*t*_(max)_ = −4.35, *p* < 0.001] and significant at left temporal electrodes [*t*_(max)_ = 3.44, *p* < 0.002, see Figure 2].

### How Much Variance in Reading Can Be Explained by the Basic Neural Measures?

Parallel to the behavioral measures, a multiple regression was run to investigate how much variance in reading fluency in fourth grade can be attributed to the neural measures recorded at the end of first grade. Overall, the three neural measures tested significantly predicted the reading outcome in fourth grade [*F*(3,47) = 4.776, *p* = 0.005, *R*^2^ = 0.234], nevertheless leaving over 70% of unexplained variance. Moreover, only the two unimodal measures of N1 print tuning (*p* = 0.004) and filtered MMN (*p* = 0.047) significantly predicted reading outcome in fourth grade, while the bimodal measure of AV congruency did not (*p* = 0.403, see Table 2). Furthermore, while higher GFP values in N1 print tuning were associated with better reading, this direction was opposite for filtered MMN as well as AV congruency (Figure 3B). For better illustration, an independent sample *t*-test was run to explore whether the neural measures tested differentiated between the lowest and the highest third of extreme readers. Only N1 print tuning significantly differentiated between the two extreme groups of readers [*t*(32) = −2.894, *p* = 0.007], while filtered MMN and AV congruency were trends [filtered MMN: *t*(32) = 1.763, *p* = 0.087; AV congruency: *t*(32) = 1.980, *p* = 0.056; see also; Figure 2].

### Can Basic Neural Measures Improve Prediction Over Behavioral Measures?

Further, we wanted to test whether basic neural measures recorded early in the course of reading acquisition (i.e., at the end of first grade) can improve prediction of the future reading outcome over behavioral measures alone. To this end, the significant behavioral predictors of RAN, block design and vocabulary were entered first, while the significant neural predictors, N1 print tuning and filtered MMN, were added in an additional block in a forward regression model. The result showed that beyond the behavioral measures of RAN, block design and vocabulary, the two unimodal neural measures of N1 print tuning and filtered MMN explained 7.2% additional variance in reading (Δ*R*^2^ = 0.072, *p* = 0.008, see also Table 3). Importantly, this combination of the behavioral and neural measures explained 57% of the entire variance in reading [*F*(5,45) = 11.982, *p* < 0.001, *R*^2^ = 0.571], suggesting that combining behavioral and neural measures can improve prediction over behavioral measures alone.

**TABLE 3:** Results of the forward regression combining the significant behavioral and neural predictors.

	Measures	*B*	*SE B*	β
Model 1	Constant	–0.68	0.51	
	RAN	–0.59	0.14	−0.50^1^
	Vocabulary	0.06	0.02	0.40^1^
	Block design	–0.03	0.01	−0.31^2^

Model 2	Constant	–1.50	0.57	
	RAN	–0.53	0.14	−0.44^1^
	Vocabulary	0.06	0.02	0.39^1^
	Block design	–0.03	0.01	−0.31^2^
	N1 print tuning	0.20	0.08	0.28^2^

Model 3	Constant	–0.75	0.60	
	RAN	–0.49	0.13	−0.41^1^
	Vocabulary	0.07	0.02	0.43^1^
	Block design	–0.03	0.01	−0.36^1^
	N1 print tuning	0.24	0.07	0.32^1^
	Filtered MMN	–0.93	0.34	−0.28^1^

*^1^*p* < 0.009, ^2^*p* < 0.05.*

## Discussion

The goal of the present study was to investigate the predictive power of behavioral and basic neural measures collected at the early stage of reading acquisition on reading outcome 3 years later. Given that the predictive value of these basic neural measures across several years were of main interest of this study, the reading measures in fourth grade were chosen as the outcome measures. Specifically, we tested whether next to the so far investigated unimodal measures of N1 print tuning and filtered MMN, the bimodal measure of audiovisual congruency effect can contribute to the prediction of the later reading outcome, and whether the neural measures can improve prediction of the later reading outcome over behavioral measures alone. RAN, block design and vocabulary were the strongest predictors, explaining over 46% of the entire variance in reading. In line with earlier studies, both unimodal measures of N1 print tuning and filtered MMN predicted reading, yet contrary to our expectation, the bimodal measure of AV congruency effect did not add to the explained variance in the later reading outcome. Most importantly, beyond the behavioral measures of RAN, block design and vocabulary, the two unimodal neural measures of N1 print tuning and filtered MMN explained 7.2% additional variance in reading.

### Rapid Automatized Naming – The Best Predictor of the Reading Outcome at the End of Fourth Grade

A considerable number of studies have identified behavioral predictors for later reading outcome (e.g., Catts et al., 2001; Schatschneider et al., 2004; Torppa et al., 2012; Brem et al., 2013). In accordance with previous literature (Compton, 2000; Manis et al., 2000; Compton et al., 2001; Wimmer and Mayringer, 2002; Lepola et al., 2005; Torppa et al., 2012; Brem et al., 2013), we found RAN to be the best predictor of the later reading outcome. Next to RAN, also phonological awareness and vocabulary have been shown to predict the later reading outcome (Wagner et al., 1997). A systematic meta-analytic review pointed out the pivotal role of phonemic awareness as a predictor of individual differences in reading development (Melby-Lervåg et al., 2012). However, in our sample of children phonological processing and auditory memory span did not contribute to the explained variance, while next to RAN, also vocabulary and block design were significant predictors of the reading outcome in fourth grade. The lack of predictive value of phonological skills in our study may be explained by three factors. First, RAN is particularly important for reading fluency, while the predictive value of phonological skills seems relatively stronger for reading accuracy and spelling rather than for reading fluency (Moll et al., 2014). Second, RAN seems to be a relatively better predictor than phonological awareness in consistent (shallow) orthographies compared to inconsistent (deep orthographies; Moll et al., 2014; Schmalz et al., 2015). Third, there are studies suggesting that phonological awareness may be a poorer long-term predictor when compared to RAN (Wagner et al., 1997; Georgiou et al., 2008).

The direction of the predictive effect of block design on reading in fourth grade was negative, meaning that children with a lower performance in the block design task showed better reading performance in fourth grade. Importantly, block design was not a significant predictor of reading in isolation, and its predictive value only became significant in combination with RAN and vocabulary. Moreover, the unexplained variance by RAN and vocabulary also correlated with the block design task. This indicates that visuospatial skills, as measured by the block design, interact with the predictive value of RAN and vocabulary.

### Unimodal Neural Measures of N1 Print Tuning and Mismatch Negativity but Not the Bimodal Measure of Audiovisual Congruency Predict Reading Outcome at the End of Fourth Grade

N1 print tuning was the most robust predictor of the later reading outcome among the neural measures. Also, previous studies emphasized the predictive value of N1 print tuning in learning to read (Bach et al., 2013; Brem et al., 2013; González et al., 2016; Soto et al., 2018). Moreover, previous studies indicated diminished sensitivity for print in young dyslexic children (Maurer et al., 2007) that may normalize with progressing reading experience (Maurer et al., 2011), but dyslexic adults still show deficient sensitivity for print (Helenius et al., 1999; Shaywitz and Shaywitz, 2005; Mahé et al., 2012). These results, together with the finding of clear structural and functional alterations in the left occipito-temporal cortex (Specht et al., 2009; Raschle et al., 2011) of preschool children with a familial risk of dyslexia and two longitudinal studies indicating the predictive power of the N1 print tuning for the later reading outcome (Brem et al., 2010; Bach et al., 2013), emphasize the importance of the potential power of print sensitivity as an index for successful reading acquisition.

A number of previous studies indicated the predictive value of auditory ERPs for language development (Molfese, 2000; Guttorm et al., 2005; Maurer et al., 2009; Choudhury and Benasich, 2011; Hämäläinen et al., 2015; Linnavalli et al., 2017). In our study, the (filtered) MMN was a significant predictor of the later reading outcome, but the negative beta-value with larger MMN associated with poorer reading contrasted results from previous studies (Maurer et al., 2009). The reason for the unexpected direction might be that the MMN was obtained only after applying a strong high-pass filter of 3 Hz that eliminated the overlapping positive mismatch response (MMR; Jost et al., 2015). A positive MMR has previously been interpreted as an immature mismatch response, as it was found in children, but not in adults (Maurer et al., 2003). It is possible that the positive MMR response was not entirely removed and that the correlation with reading skills may be driven by the original (immature) positive MMR rather than the filtered MMN. Although the correlation between positive MMR and fourth grade reading was not significant (*r* = −0.075, *p* = 0.600), a group contrast between good and poor readers showed a nominally larger MMR for the good readers, supporting the idea that the correlation between filtered MMN and reading may be driven by an incompletely removed positive MMR (see Supplementary Materials A1 and F1). Moreover, the effect of the filtered MMN on later reading was rather weak, as it only occurred together with the other predictors, but not when added as a single predictor.

As previous studies indicated the crucial role of the integration of auditory and visual linguistic inputs for reading (Ehri, 2005; Blau et al., 2009, 2010; Blomert, 2011), this study aimed at investigating whether AV congruency effects could predict reading better than the so far investigated unimodal measures on N1 print tuning (Bach et al., 2013; Brem et al., 2013; Eberhard-Moscicka et al., 2014) and MMN (Maurer et al., 2009). To date, this question has been addressed by a single study with a smaller sample size that used artificial-letter training (Karipidis et al., 2018). However, unlike the previous study, we did not find any significant prediction of later reading skills by the AV congruency effect around 200 ms. Moreover, the group contrast suggested a larger AV congruency effect for poor readers than good readers, contrasting previous results that showed larger congruency effects for typically reading children (Blau et al., 2010) and adults (Blau et al., 2009), but not for their dyslexic peers. Moreover, a larger AV congruency effect was found in children who became good readers compared to those who became poor readers (Karipidis et al., 2018). As the time window selected in our study (180–196 ms) was earlier than in the study by Karipidis et al. (2018), the neural processes measured may reflect different aspects of audiovisual integration. We therefore performed an additional analysis (see Supplementary Material F2) with the STEN toolbox (Knebel and Notter, 2018) that indicated a second, later time window (late AV congruency: 544–560 ms). As such, parallel to the main analysis, an additional analysis was run in order to investigate whether the later time window of the AV congruency effect would yield a significant result. Again, the (late) AV congruency effect did not predict later reading (see the Supplementary Material A2), even though the effect tended to be larger in good readers compared to poor readers (see Supplementary Material F2), similar to previous studies (Blau et al., 2009, 2010; Karipidis et al., 2018).

Importantly, in accordance with the main analysis, also the multiple regression analysis with the (late) AV congruency effect indicated that only the two unimodal measures of N1 print tuning (*p* = 0.002) and filtered MMN (*p* = 0.039) but not the bimodal measure of (late) AV congruency effect (*p* = 0.873) were predictive of the future reading outcome (see Supplementary Material T2). Yet, these results do not generally contradict the notion of letter-sound integration constituting an emergent property of learning to read (Blau et al., 2009, 2010). They may rather suggest that first grade might be too early to study multisensory integration processes at the word level; and/or that audiovisual integration at this stage might be more basic, and AV integration effects at the level of letters and phonemes may be better predictors of reading acquisition (Karipidis et al., 2018). It also seems plausible that neural processes underlying audiovisual integration of words may become more important predictors later on during reading acquisition.

### Basic Neural Measures Can Improve Prediction of the Future Reading Outcome Over Behavioral Measures Alone

We found that N1 print tuning and filtered MMN improve prediction of the future reading outcome over behavioral data alone. This is in line with previous studies that showed improved prediction of reading development, if neural measures were added to behavioral measures (Hoeft et al., 2007; Maurer et al., 2009; Brem et al., 2013). While the current findings confirm the results of previous investigations (Brem et al., 2013) by showing that N1 print tuning explains additional variance of future reading skills, the current results also extend those previous studies by showing that N1 print tuning has predictive value not only before the start of formal schooling but also in the first phase of learning to read at school. Moreover, the results of the current study suggest that MMN measures potentially add explanatory power for predicting reading skills, although the underlying processes measured in the current study may rather be tied to an immature mismatch response than to processes tapped in previous studies. Taken together, these results indicate the potential value of combining measures from different methods (i.e., neural and behavioral) to advance prediction of the future reading outcome. This predictive value of the neural measures shall be of particular importance in preliterate children, where behavioral measures are typically of limited applicability. A practical implementation may entail development of targeted intervention programs that may include, yet are not limited to, grapheme–phoneme trainings that can be applied early in the course of development, as has been demonstrated by, e.g., Karipidis et al. (2017) with kindergarten children.

## Conclusion

To our knowledge, no study to date has combined visual, auditory and audiovisual neural measures together with behavioral measures to investigate their predictive value for later reading skills in a larger sample of children. Although these results shall be interpreted with caution, this study provides important information on the predictive power of the basic neural and behavioral measures and that the neural measures can improve prediction over behavioral measures alone.

## Data Availability Statement

The datasets generated and analyzed during the current study are available from the corresponding author on reasonable request. Requests to access the datasets should be directed to AE-M, aleksandra.eberhard@neuro.unibe.ch.

## Ethics Statement

The studies involving human participants were reviewed and approved by University of Zurich Ethics Commission, Faculty of Arts and Social Sciences, Zurich. Written informed consent to participate in this study was provided by the participants’ legal guardian/next of kin.

## Author Contributions

AE-M, LJ, and UM contributed to the material preparation, data collection, and performed the analyses. AE-M wrote the first draft of the manuscript. All authors contributed to the study conception, design, and commented on previous versions of the manuscript. All authors read and approved the final manuscript.

## Conflict of Interest

The authors declare that the research was conducted in the absence of any commercial or financial relationships that could be construed as a potential conflict of interest.

## Publisher’s Note

All claims expressed in this article are solely those of the authors and do not necessarily represent those of their affiliated organizations, or those of the publisher, the editors and the reviewers. Any product that may be evaluated in this article, or claim that may be made by its manufacturer, is not guaranteed or endorsed by the publisher.
