# Supplementary material for: Predicting Reading From Behavioral and Neural Measures – A Longitudinal Event-Related Potential Study
Source: Front Psychol. 2021 Nov 30;12:733494. doi: 10.3389/fpsyg.2021.733494 (PMC8669350; doi:10.3389/fpsyg.2021.733494)
Supplement: Supplementary file 1 [file Data_Sheet_1.pdf]

Supplementary Table 1: T1 and collinearity indices for the behavioral measures.

T1. Correlation matrix of the behavioural measures tested in 1st grade

MEASURES	Reading fluency	RAN	Phonological processing	Vocabulary	Block design
Reading fluency	___				
RAN	-.416[1]	___			
Phonological processing	.304[2]	-.416[1]	___		
Vocabulary	.413[1]	-.363[1]	.386[1]	___	
Block design	.225	-.312[2]	.418[1]	.521[1]	___
Auditory memory span	.322[2]	-.336[2]	.403[1]	.263[1]	.386[1]

[1]$p<.005$; [2]$p<.05$

There was no multicollinearity between the behavioral measures as all the correlation coefficients were well below .80 (all r < .522), the variance inflation factors were below 2 (all VIFs < 1.545), and the tolerance scores were above 0.6 (all Tolerance scores > .647).

Supplementary Analysis on the mismatch response MMR: A1.

An additional analysis was performed on the unimodal auditory neural measure using standard filter settings (i.e., 30Hz low-pass and .3Hz high-pass). In accordance with the main analysis, the time window of interest for the MMR was set to a length of 16ms and was based on the GFP peak of the difference between deviant "ta" and standard "da" stimuli, resulting in a time window of 224-240ms.

Supplementary Figure on the mismatch response MMR (222-240ms): F1.

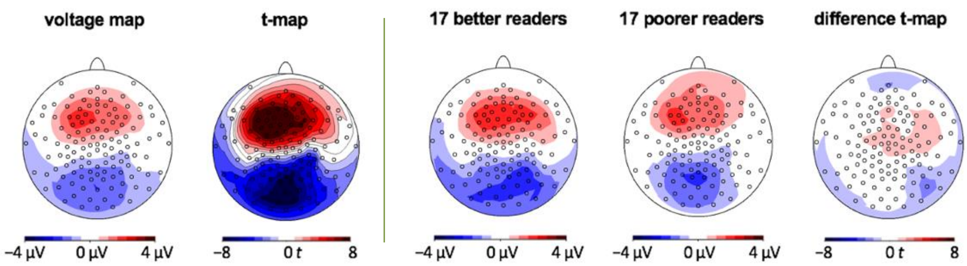

F1. The left part of the figure depicts a voltage map and a corresponding *t*-map of the MMR segment, while the right part of the figure depicts voltage maps and a corresponding difference *t*-map for the lowest third of poorer and the highest third of better readers.

Supplementary Analysis on the late AV congruency effect: A2.

A supplementary analysis on the late AV congruency effect was run in order to investigate whether the chosen time window of 180-196ms was not too early to detect the AV congruency effects. In accordance with the main analysis, the time window of interest was set to a length of 16ms and was based on the GFP peak of the difference between AVN and AVM stimuli. Parallel to the main analysis, a multiple regression was run to investigate how much of the entire variance in reading

fluency in 4th grade can be attributed to the neural measures recorded at the end of 1st grade. Similar to the main analysis, the neural measures tested significantly predicted the reading outcome ($F(3,47)=4.482$, $p=.008$, $R^2=.222$). Nevertheless, only the two unimodal measures of N1 print tuning ($p=.002$) and filtered MMN ($p=.039$) significantly added to the prediction, while the bimodal measure of late AV congruency did not ($p=.873$, see T2).

Supplementary Figure on the late AV congruency effect: F2.

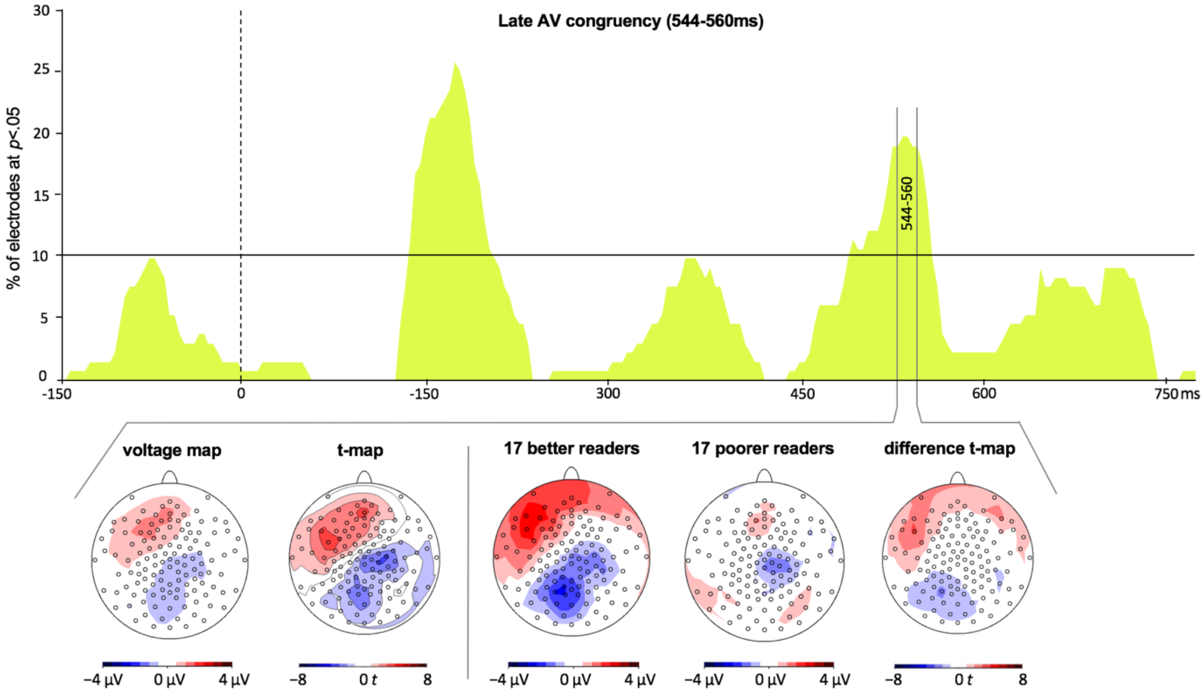

F2. Electrode-wise, time-wise paired *t*-tests indicating significant differences between AVN and AVM stimuli (at $p<.05$, for at least eight consecutive timeframes, i.e., 32ms). While this time-wise analysis over the whole electrode montage is highly sensitive to detect ERP modulations, it entails a large number of statistical tests and is thus prone to detect false-positive results. In order to correct for multiple comparisons and for temporal and spatial auto-correlation, only periods showing differences for at least 32ms on more than 10% electrodes (i.e., the solid horizontal line; see e.g. Guthrie and Buchwald, 1991; Hartmann et al., 2016; Murray et al., 2008; Sallard et al., 2018 for corresponding approaches) were considered in this analysis. The lower panel of the figure depicts a voltage map and a corresponding *t*-map of the late AV congruency segment as well as voltage maps and a corresponding difference *t*-map for the lowest third of poorer and the highest third of better readers.

Supplementary Table 2: T2.

T2. Multiple regression analysis (method enter).

MEASURES	Reading fluency (4th grade)	*B*	*SE B*	*β*
Neural (1st grade)	Constant	-.29	.60	
	N1 print tuning	.31	.10	.42[1]
	Filtered MMN	-.92	.43	-.28[2]
	Late AV congruency	-.03	.16	-.02

[1]$p<.005$, [2]$p<.05$

References

Guthrie, D., Buchwald, J.S. (1991). Significance testing of difference potentials. Psychophysiology. 28(2), 240-244. doi.org/10.1111/j.1469-8986.1991.tb00417.x

Hartmann, L., Sallard, E., Spierer, L. (2016). Enhancing frontal top-down inhibitory control with Go/NoGo training. Brain Struct Funct. 221, 3835-3842. doi.org/10.1007/s00429-015-1131-7

Murray, M.M., Camen, C., Spierer, L., Clarke, S. (2008). Plasticity in representations of environmental sounds revealed by electrical neuroimaging. NeuroImage. 39, 847-856. doi.org/10.1016/j.neuroimage.2007.09.002

Sallard, E., Hartmann, L., Ptak, R., & Spierer, L. (2018). Spatiotemporal brain dynamics underlying attentional bias modifications. Int J Psychophysiol.130, 29-39. oi.org/10.1016/j.ijpsycho.2018.06.001
